# Envemind: Accurate Monoisotopic Mass Determination Based On Isotopic Envelope

**DOI:** 10.1021/jasms.2c00176

**Published:** 2022-10-12

**Authors:** Piotr Radziński, Dirk Valkenborg, Michał Piotr Startek, Anna Gambin

**Affiliations:** † Institute of Informatics, University of Warsaw, 00-927 Warsaw, Poland; ‡ Interuniversity Institute of Biostatistics and Statistical Bioinformatics, Hasselt University, BE3500 Hasselt, Belgium; § Institute of Immunology, University Medical Center of the Johannes-Gutenberg University Mainz, 55131 Mainz, Germany

**Keywords:** monoisotopic mass, linear model

## Abstract

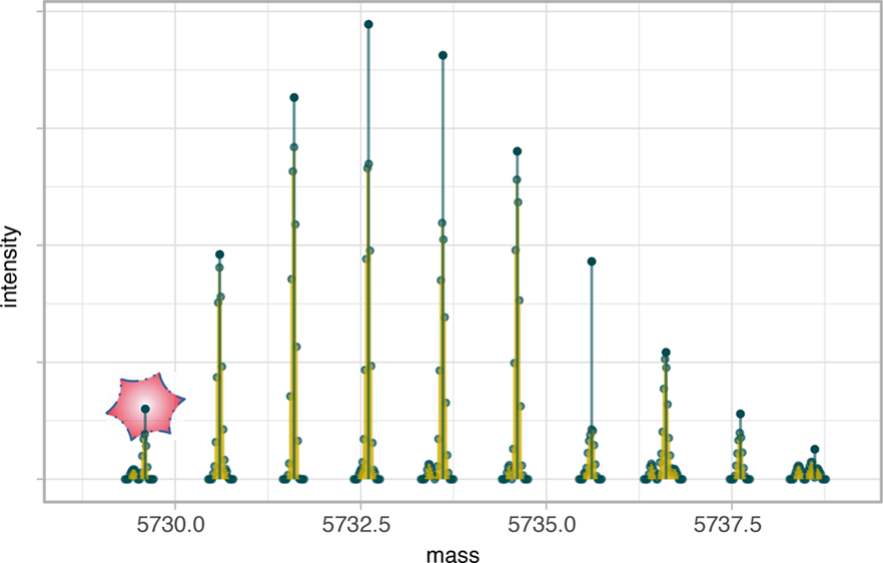

Nowadays, monoisotopic mass is used as an important feature in top-down proteomics. Knowing the exact monoisotopic mass is helpful for precise and quick protein identification in large protein databases. However, only in spectra of small molecules the monoisotopic peak is visible. For bigger molecules like proteins, it is hidden in noise or undetected at all, and therefore its position has to be predicted. By improving the prediction of the peak, we contribute to a more accurate identification of molecules, which is crucial in fields such as chemistry and medicine. In this work, we present the envemind algorithm, which is a two-step procedure to predict monoisotopic masses of proteins. The prediction is based on an isotopic envelope. Therefore, envemind is dedicated to spectra where we are able to resolve the one dalton separated isotopic variants. Furthermore, only single-molecule spectra are allowed, that is, spectra that do not require prior deconvolution. The algorithm deals with the problem of off-by-one dalton errors, which are common in monoisotopic mass prediction. A novel aspect of this work is a mathematical exploration of the space of molecules, where we equate chemical formulas and their theoretical spectrum. Since the space of molecules consists of all possible chemical formulas, this approach is not limited to known substances only. This makes optimization processes faster and enables to approximate theoretical spectrum for a given experimental one. The algorithm is available as a Python package envemind on our GitHub page https://github.com/PiotrRadzinski/envemind.

## Introduction

According to a recent overview,^1^ top-down proteomics has been transitioning toward clinical research. This change in focus has been accomplished by sample preparation and cleanup improvements. Furthermore, technologies for separating intact proteins bring the characterization of global proteoforms in a range of top-down proteomics, while advancements in mass spectrometry instrumentation have enabled the characterization of large proteoforms in complex mixtures. Nonetheless, despite the aforementioned improvements, some open problems and hurdles still exist to be taken in the bioinformatic analysis of top-down proteomics data. A case in point is the effective and accurate determination of the precursor mass of the unknown proteoforms, as this will reduce the ambiguity of identification in a database search. However, as already mentioned by Lermyte et al.,^2^ the concept of *precursor mass* needs some reconsideration, as the isotope peaks related to the intact proteoforms lead to the occurrence of broad, complex isotope distributions that do not unveil the accurate monoisotopic mass.^3^ As such, the monoisotopic and average masses and the mass of the most abundant (aggregated) isotope peak are possible candidates to determine the precursor mass. To date, the default mass reported in top-down experiments is the average mass, which is also experimentally the most easily accessible value from both resolved as unresolved isotope distributions in case the resolving power is insufficient to separate the isotope peaks. However, as indicated by Claesen et al.,^4^ this average value is sensitive to natural and technical variations. The reason is that uncertainty at the level of the elemental isotope definition or variation in the spectral accuracy will creep into the equation to compute the average mass values. The consequence is that the ambiguity in the protein identification will increase in a database search because of the larger search tolerance set on the precursor mass. On a more positive note, the average mass is a metric that can be easily computed for databases and is interoperable among the various top-down bioinformatics tools.

In an ideal situation, one would opt for the monoisotopic precursor mass, as this value does not depend on the elemental isotopic abundances nor spectral accuracy. This metric is also easy to compute for databases and is interoperable with other bioinformatics tools, but unfortunately, the probability of occurrence of the monoisotopic variant is extremely low for intact proteins. Therefore, the monoisotopic variant of the proteoform falls below the detection limit and is not observed in a mass spectrum.

As argued by Claesen et al., a good alternative that strikes a balance between the ease of detection and reduction of ambiguity would be to use the mass of the most abundant isotope peak. The uncertainty hardly influences this mass value in the elemental isotopic abundances, and there is a robust solution that can prevent interference from low spectral accuracy. Unfortunately, the mass of the most abundant aggregated isotope peak is more difficult to compute, as it requires a computer algorithm like, for example, BRAIN(5) or IsoSpec,^6^ as opposed to the monoisotopic and average mass, which can be obtained instantly from a chemical formula. Further, the most abundant mass is not interoperable with other proteomics software suites and, therefore, is never considered a viable alternative.

This conundrum was solved by Senko et al.^7^ who proposed to use an averagine-scaling method that searches for a scaled averagine molecule for which its theoretical isotope distribution best fits the observed isotope distribution in the spectrum. The scaled averagine molecule acts as a surrogate for the observed molecule, and the monoisotopic mass can be computed from the atomic composition of the obtained scaled averagine. This method works very well and is still used in many software packages. However, the procedure entails a dynamic fitting procedure that can be demanding for computers, given the high-throughput nature of current top-down proteomics experiments.

In the search for an alternative and more static strategy, Dittwald et al.^8^ proposed a linear model to predict the monoisotopic mass based on the observed most abundant isotope peak. A concept was further explored and improved by Chen et al.,^9^ who serendipitously uncovered a linear correlation between these two protein masses (sic.). Finally, Lermyte et al.^2^ developed the MIND algorithm that entails a double linear model to predict the monoisotopic mass from partially observed isotope patterns along with a robust selection method to determine the theoretical most abundant peak mass under a poor ion statistic. This strategy combines the best of both worlds and allows for an interoperable metric that can be robustly predicted from the partial isotope distribution.

An open problem in the monoisotopic mass prediction is the off-by-one dalton error also present in the MIND application. According to the MIND manuscript, the off-by-one errors appear on average in 31.9% of proteins over the specified mass range, but for heavier proteins, this error can become more abundant and leads to wrong predictions in 51% of the predicted cases. This problem needs attention. Therefore, we present an algorithm that relates observed isotope distribution to the monoisotopic mass of a protein while it aims at reducing this infamous off-by-one dalton error for high-resolution spectra. The procedure is composed of three steps. A first step is inspired by the averagine scaling of Senko et al. The second step is a first predictor inspired by the MIND algorithm but with an additional predictor variable that captures information about the width of the isotope distribution. The third step is an optimization procedure that searches for the monoisotopic mass with the highest likelihood over a fine-grained grid. Linear models in our method are trained on proteins with an 8–400 kDa mass range; however, the envemind algorithm was also successfully tested on smaller proteins. The method is compared against our MIND algorithm and outperforms MIND on simulated data in terms of reducing the off-by-one dalton error.

## Methods

### Envemind Algorithm

**Figure 1:**
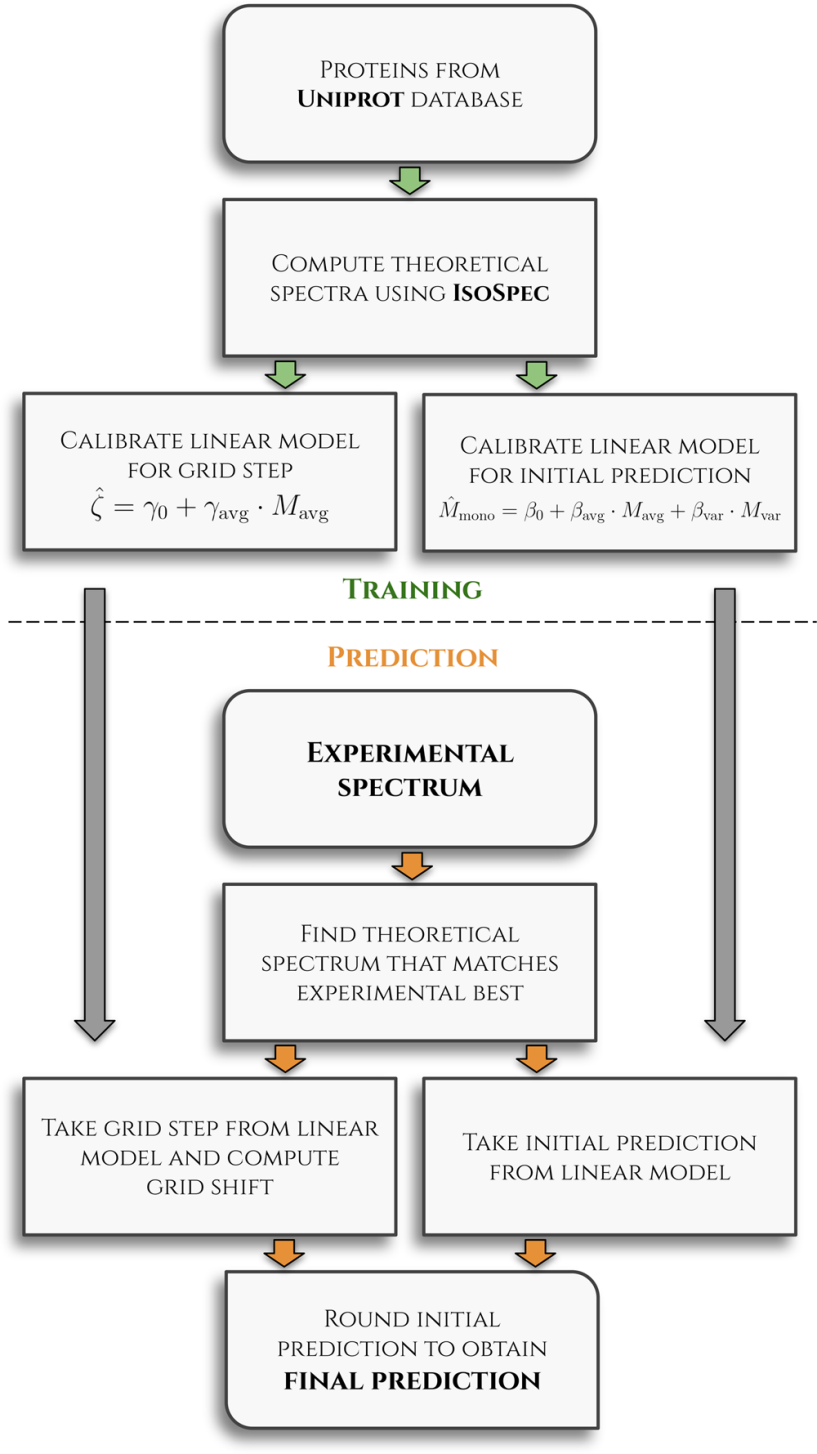
Control flow graph illustrating methodology of the envemind algorithm. It is divided into two parts. First, *training*, with calibration of linear models on theoretical spectra, and second, *prediction*, which deals with an experimental spectrum by a fitting procedure and linear models from the previous part.

The procedure of monoisotopic mass determination is divided into two parts. In the beginning, we look for a theoretical spectrum that best fits a given experimental one. By *theoretical spectrum*, we mean a simulated spectrum without any noise, equivalent to the probability mass function of multinomial distribution with atomic masses as values and natural isotopic abundances as probabilities.^10^ Having the theoretical spectrum simplifies the prediction problem. It allows us to build a mathematical model that requires precisely measured features, usually unavailable in experimental spectra (like variance), which, calibrated on theoretical spectra, provide accurate predictions. To simplify the presentation, we begin with a description of prediction on theoretical spectra, and then we describe how to deal with experimental ones. More technical details are moved to the Supporting Information. A control flow graph through envemind containing the main steps of the algorithm is presented in Figure 1.

### Theoretical Spectra

In this part, we describe the prediction of the monoisotopic mass *M*_mono_ for a given theoretical spectrum. Hence, all computations and calibrations presented in this section are done on theoretical spectra only. The spectra were simulated by IsoSpec based on chemical formulas randomly chosen from Uniprot database^11^ with an 8–400 kDa mass range. In this section, the theoretical spectra *x*-axis contains daltons. Therefore, all parameters described are also in daltons. We first take simple initial predictions from the linear model that uses the average mass of a protein *M*_avg_ and the variance of its spectrum *M*_var_. The following linear model has been trained and tested on approximately 1.9 million spectra:
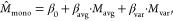
1with fitted coefficients β_0_ = −0.145 57, β_avg_ = 0.999 78, and β_var_ = −0.598 17. For some proteins, the outcome of this linear predictor (1) is erroneously shifted. However, the 10-fold cross-validation yields the absolute error of prediction below 0.5 Da for ca. 96.6% of proteins. The next step is intended to reduce this bias by predicting a grid of possible locations of monoisotopic mass. Recall that isotopologues in spectra are aggregated into clusters separated by ca. 1 Da. Therefore, the determination of such a grid enables us to round *M̂*_mono_ to the closest point on the grid. Let us define the grid as follows:
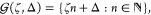
where ζ is a distance between nodes of the grid, and Δ is a shift of the grid.

#### Estimation of the Grid Step ζ

**Figure 2:**
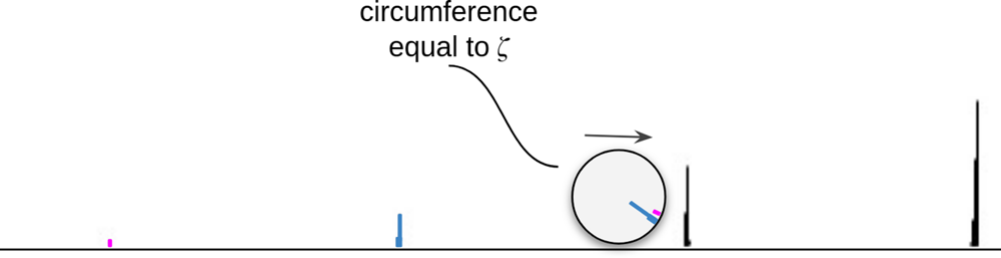
Circle that rolls across spectrum. For ζ that estimates well the distance between groups of peaks, peaks transformed into the circumference of a circle should overlap on a small fragment.

The purpose of the ζ parameter is to control the spacing between peak clusters. To determine the distance between two consecutive grid nodes, consider the circle rolled through the protein’s spectrum like a glue-coated roller that collects all peaks. The ideal length of the grid step (circumference of a circle ζ) will make all the peaks stick in a small section of the circle, cf. Figure 2. If all the isotope peaks can be collected and encapsulated in a small region on the sticky roller, then the circumference of the circle is equal to the average distance between consecutive isotope clusters.

More formally, the sticky roller procedure described above transforms all peaks in the spectrum , that is, pairs *p* = (*p*^mass^, *p*^prob^), to complex unit circle, rotates them to average zero (to avoid problems with logarithm specification on complex plane), and then transforms to the interval [−ζ/2, ζ/2]. The final transformation looks as follows:
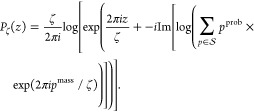


To quantify the concentration of peaks, we treat the spectrum  as a random variable and make use of the notion of variance. Therefore, the optimal ζ minimizes the variance of the transformed spectrum:
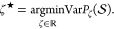


The variance minimization procedure requires a few minutes of computation for the average-size proteins, and this time grows exponentially for bigger molecules, which can hinder practical applications. For this reason, we propose the universal grid step ζ̂ based on the observation that ζ^★^ is slightly correlated with a protein’s average mass (correlations: Pearson: 0.12, Kendall: 0.07, Spearman: 0.11). Therefore, we adjusted a linear model ζ^★^ ∼ *M*_avg_. The linear model for the universal grid step is as follows:
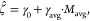
2with coefficients γ_0_ = 1.002 355 and γ_avg_ = 6.9584 × 10^–10^. For future use let us note that, since γ_avg_ is very small, measurement error of *M*_avg_ leads to negligible change of ζ̂. Therefore, we can also compute ζ̂ based on the experimental spectrum’s average mass. More details are given in the Supporting Information.

#### Estimation of the Grid Shift Δ

Once we have chosen ζ̂, we can focus on the grid shift parameter Δ. In this setting, the Δ matches the spectrum best if it minimizes the distance between grid points and spectrum peaks, that is, can be formulated as:
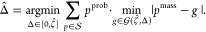
However, the computations can be accelerated by transforming the spectrum to a complex unit circle and finding the mean point in the complex space:
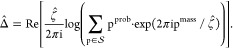
Both approaches return the same Δ̂. The whole procedure is efficient enough to calculate grid shift for each protein independently.

#### Final Prediction

**Figure 3:**
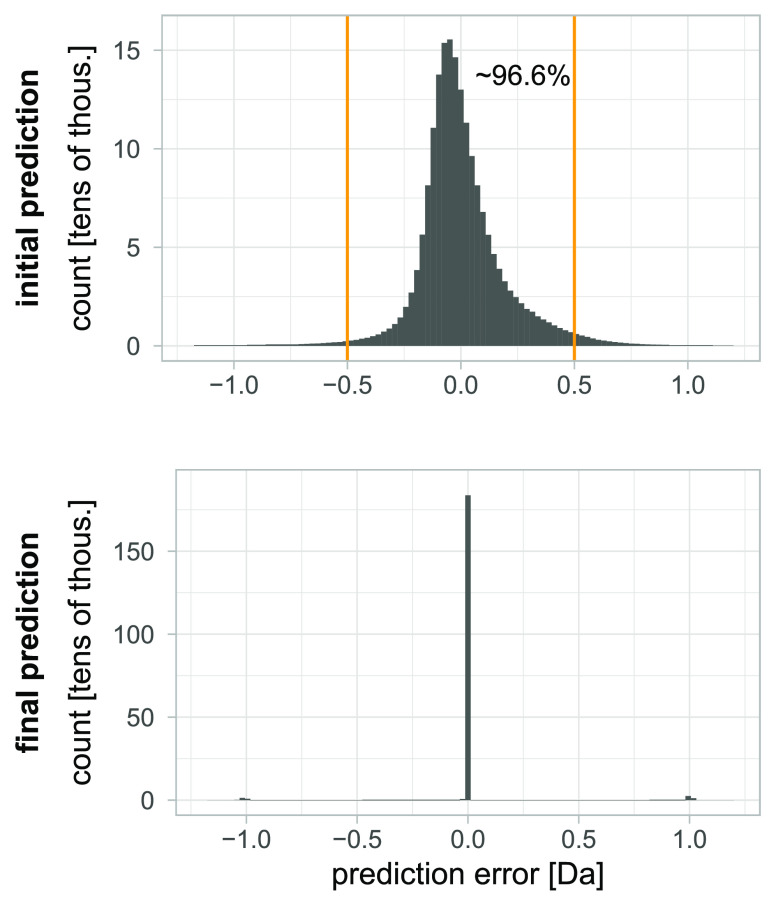
Comparison between the linear model’s initial prediction and the final prediction made by adjusting the grid and rounding the initial prediction to the closest point on it. Orange lines designate an interval inside which predictions will round to the most appropriate point on the grid .

Recall that, according to the described procedure, the final step in prediction of monoisotopic mass *M*_mono_ is to round the initial prediction *M̂*_mono_ to the closest point on the grid . However, it turns out that the distances between clusters of aggregated peaks in a given spectrum are not perfectly equal. When one considers the distribution of intercluster distances centered on the most abundant peak, those in the left tail (for smaller masses) tend to increase slightly. It makes the distribution of errors after rounding a bit shift from zero. As a remedy, we added the term λ that centers the distribution of errors to have an expected value equal to 0, which results in a slightly better prediction. Technical details on how the shift was constructed can be found in the Supporting Information. Summarizing, the final prediction model reads as follows:
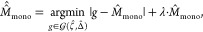
where λ = −1.1982 × 10^–7^. Distributions of errors for initial and final predictions are presented in Figure 3.

### Experimental Spectra

We expand the described model for theoretical spectra to work with experimental ones. First, in this section, we fix the experimental spectrum denoted by . Notice that one of the essential predictors was the variance of the spectra. Unfortunately, having an exact variance value for experimental spectra is hardly possible. Therefore, we would like to construct a theoretical spectrum similar to the true theoretical spectrum of an experimental spectrum’s substance. Also, in this section, spectra are assumed to contain daltons on the *x*-axis. If a given experimental spectrum would be *m*/*z*, then the preliminary step is recalculating it into daltons.

Our construction of the simulated spectrum is based on the concept of *averagine* proposed by Senko et al.^7^ but is far more complex. We define averagine as a hypothetical molecule with chemical formula
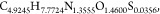
with the average mass equal to 110.4728 Da. We followed the methodology of Senko’s work to obtain the formula on Uniprot database, which is much bigger than in the original work. Therefore, the new averagine formula is slightly different from the old one.

Senko proposed an algorithm to approximate proteins’ chemical formulas, from which monoisotopic mass can be easily calculated. The algorithm follows: take multiplied averagine to obtain an average mass equal to the experimental spectrum’s average mass, round the formula to integers, and add hydrogen to be close to the experimental spectrum’s average mass again. Nowadays, using IsoSpec,^6^ we can efficiently simulate the theoretical spectrum of any chemical formula. Therefore, we consider the 5-dimensional space of protein chemical formulas, where every dimension corresponds to the number of atoms of a molecule’s chemical element (C, H, N, O, and S). In such a vast space, we would like to account not only for the mass of the protein but also for the different possible shapes of its isotopic envelope. Hence, we modify Senko’s approach.

**Figure 4:**
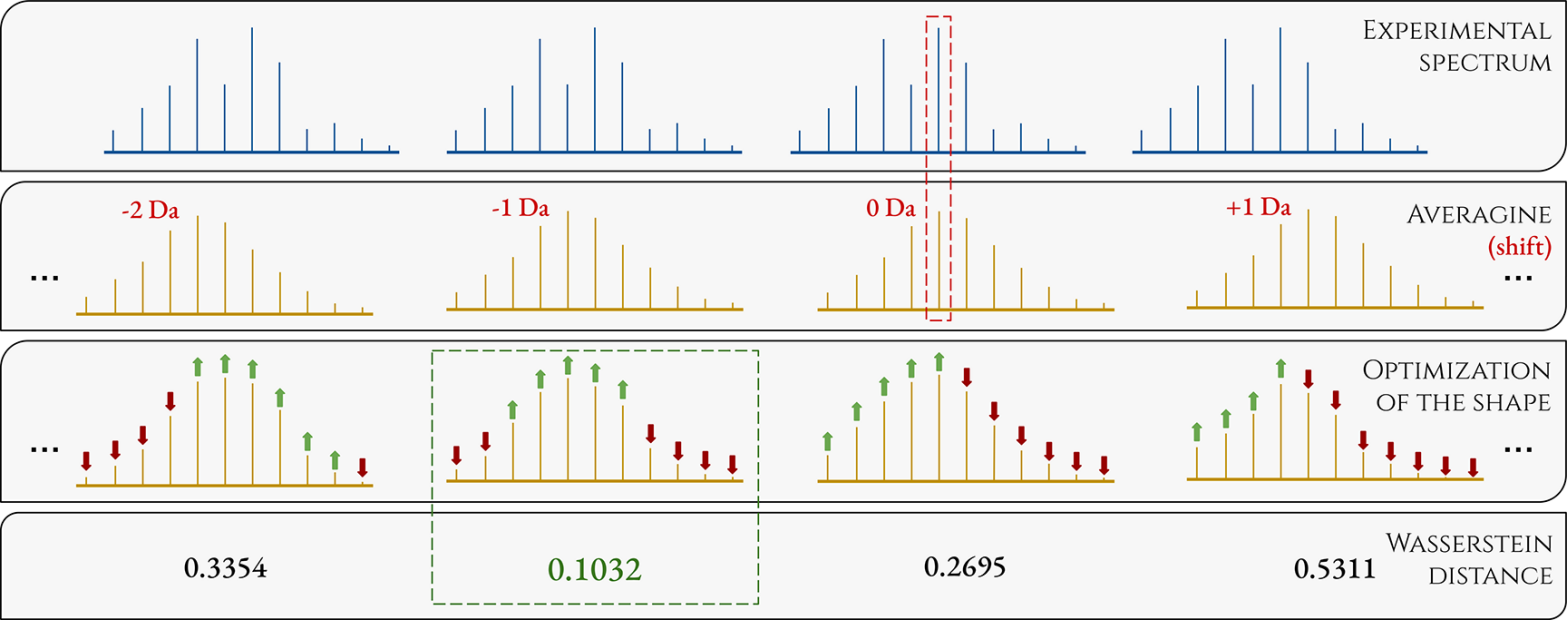
Theoretical (simulated) spectrum searching scheme for a given experimental one. In the first row, copies of the experimental spectrum are presented. The row below shows copies of averagine with different shifts. For example, a shift by 0 Da means that the most abundant aggregated peak of the simulated spectrum is in the place of the most abundant peak of the experimental spectrum (red frame). Then, we optimize variance for every averagine copy separately due to Wasserstein distance to the experimental spectrum. Arrows show if intensities grow or decrease due to the optimization. Finally, the spectrum with the lowest score will be used to further prediction by linear models (green frame).

We suggest keeping an eye on Figure 4 when reading the following description, as it should be helpful. Let us define spectrum  with two parameters.  is Senko’s averagine shifted this way, that its first aggregated peak to the left of its average mass is in the place of most abundant peak of  (red frame in Figure 4).

The parameter *k* is responsible for a shift of the spectrum and means that spectrum is shifted by *k* · ζ̂ Da. To hold peaks in proper places, we keep *k* as an integer. This way, we can easily obtain copies of averagine shifted to different locations, but we are sure that the peaks of the simulated spectra are in locations similar as experimental ones. An example of such shifted copies of averagine can be seen in the second row of Figure 4.

The parameter ρ is responsible for variance change. We developed a formula that, by adding it to a given molecule, changes the variance of the molecule’s spectrum in the fastest possible way but does not change its average mass (see Supporting Information for details). The formula should be added after checking how many averagine molecules can be contained in a given mass, but before the rounding. Parameter ρ is a multiplier of the formula and controls how much the simulated spectrum’s variance should be modified.

**Figure 5:**
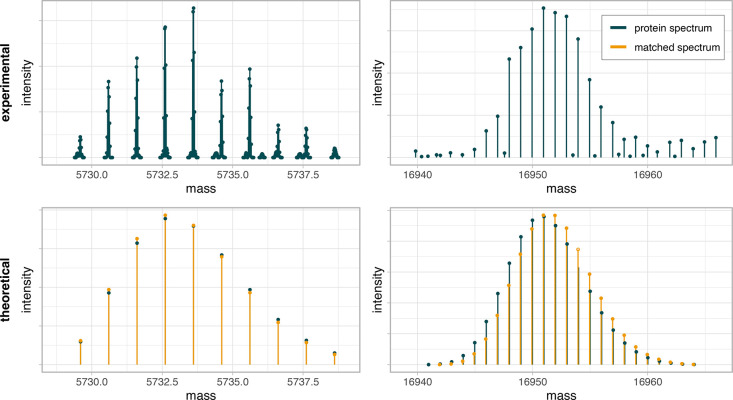
Two examples of matched spectra. In each column, experimental and theoretical spectra of insulin and myoglobin are presented, respectively (blue). In the second row, theoretical spectra that were matched (simulated) to the experimental one are imposed on (orange). Note that, in the right column, we present a badly matched spectrum for which off-by-dalton error occurred.

We have to optimize those two parameters to obtain the final simulated spectrum. We use Wasserstein distance *W*(12,13) to compare spectra, which is fast to compute and gives relatively good comparison scores. Note that, for every fixed shift *k* of initial averagine, a different parameter value ρ will minimize the Wasserstein distance. Therefore, for a given *k* the optimal quantity of ρ can be formulated as
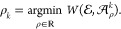
Then, concerning optimal values of ρ_*k*_, we can optimize the shift of the spectrum
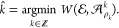
Finally,  is the spectrum we look for. In simpler words, we optimize the variance separately for different copies of averagine shifted by *k* · ζ̂ Da and then pick the spectrum with the smallest Wasserstein distance to the experimental spectrum . We use the spectrum to run the prediction described in the previous *Thoeretical spectra* section to obtain monoisotopic peak mass. Examples of constructed spectra with their theoretical and experimental substitutes can be found in Figure 5.

### Data

To train and test linear models on theoretical spectra, we took proteins from the Uniprot database^11^ with 8–400 kDa mass range; other proteins were omitted due to its minority. From a chemical formula, we computed a protein’s theoretical spectrum using IsoSpec isotopic structure calculator. The smallest peaks were not computed to keep computational time low, and the algorithm stops when the summed intensity (probability) exceeds 99%. Both linear models (1) and (2) were cross-validated on 1.9 million and 80 000 randomly chosen proteins, respectively.

We used the same spectra as in MIND for the proof-of-concept experiments. Spectra were acquired on a Thermo LTQ OrbitrapVelos, operated at a resolving power of 10^5^ at 400 *m*/*z*, and 10^6^ charges were accumulated in the LTQ for analysis in the Orbitrap. Immediately prior to infusion of the protein, external calibration was performed via an automatic routine, using a standard calibration mix containing *n*-butylamine, caffeine, MRFA, and Ultramark 1621 (PierceLTQ Velos ESI Positive Ion Calibration Solution, Thermo catalog no. 88323). Bovine insulin (Sigma catalogue no. I5500; 50 scans with 3 well visible charges; monoisotopic mass 5729.60 Da, average mass 5733.58 Da), equine apo-myoglobin (Sigma catalogue no. M0630; 200 scans with 2 well visible charges and another 100 scans with 3 well visible charges; monoisotopic mass 16 940.97 Da, average mass 16 951.50 Da), and equine cytochrome c (Sigma catalog no. C2506; 400 scans with 3 well visible charges; monoisotopic mass 12 352.23 Da, average mass 12 360.21 Da) were acquired from Sigma and infused at a concentration of 1 μM in 49:50:1 H_2_O/MeCN/HCOOH, without further purification, using nano-ESI (ESI = electrospray ionization) with an Advion Triversa Nanomateinlet system. In summary, we possess 550 (150 bovine insulin and 400 equine apo-myoglobin) high-resolution experimental spectra segments (i.e., selected intervals with a single charge in it) and 1500 (300 equine apo-myoglobin and 1200 equine cytochrome c) lower-quality spectra segments.

## Results and Discussion

Let us remind that the envemind algorithm is dedicated to spectra with quality good enough to easily distinguish an isotopic envelope “by eye”. Therefore, we divided testing into three categories of spectra quality. First, simulated spectra that were computed by IsoSpec based on chemical formulas of actual proteins from the Uniprot database. The remaining two categories are experimental spectra: with visible isotopic envelope and somewhat noisy. Finally, we compared our results to those of the MIND algorithm for which prediction is based on the most abundant peak. For experimental spectra, we preprocessed data to select the reliable most abundant peak as described in their work and then with use of an online shiny app (https://valkenborg-lab.shinyapps.io/mind/).

**Figure 6:**
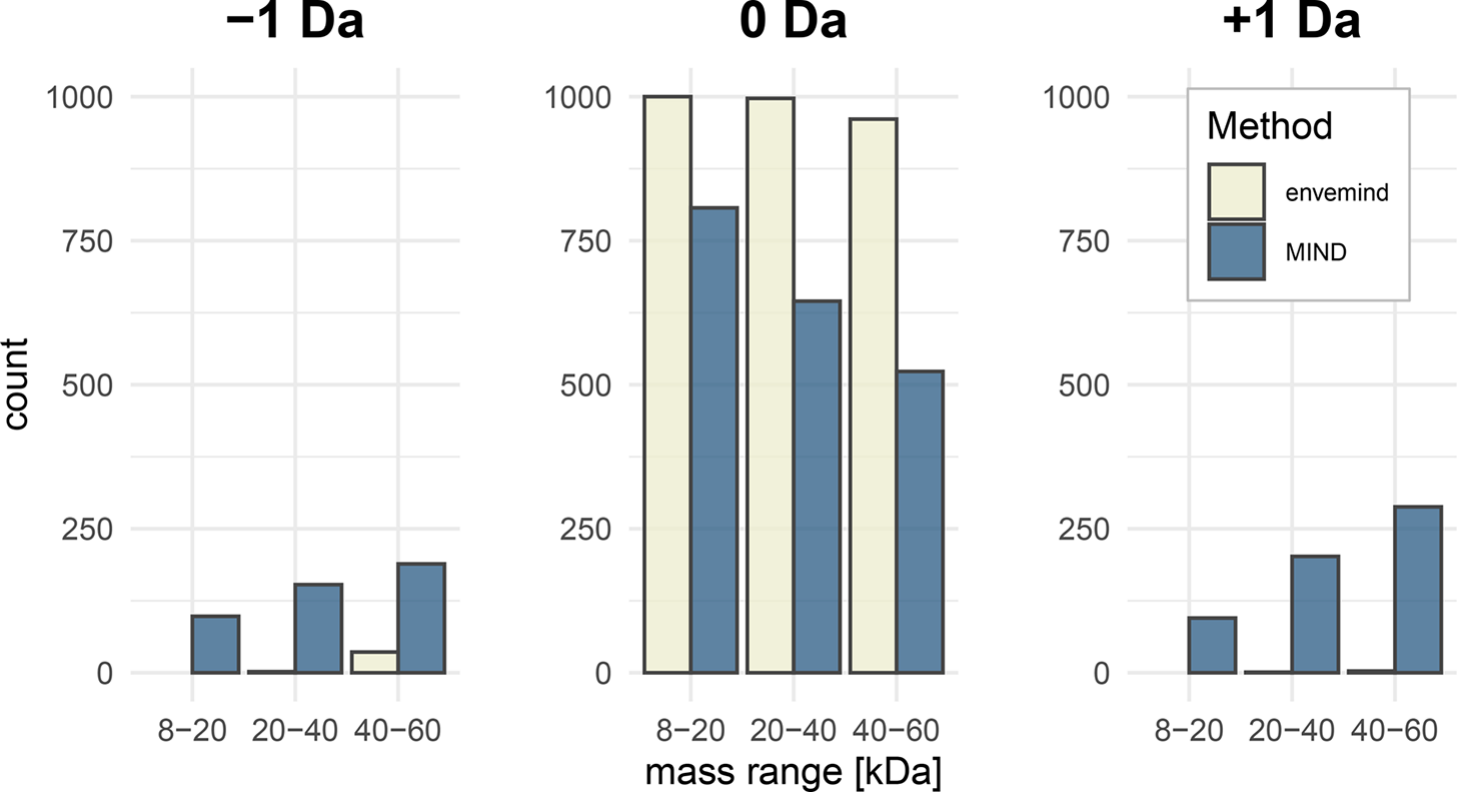
Comparison to find how often off-by-one dalton errors occur for envemind and MIND algorithms. Tests were performed on theoretical spectra of 1000 randomly chosen proteins in three mass range groups. If the prediction absolute error is ca. 2 Da or more, we include the error to responding ±1 Da bar.

We begin with tests on simulated spectra. Since the MIND algorithm was trained on proteins within the 8–60 kDa mass range, first, we tested envemind on a wider range of 8–400 kDa. The mean absolute error (MAE) of monoisotopic mass prediction was 0.51 ppm (0.0358 Da). For 96.6% of proteins, off-by-one dalton errors did not occur. For them, MAE was 0.0526 ppm (0.0020 Da). To compare with MIND, we ran the prediction in three mass range groups: 8–20, 20–40, and 40–60 kDa. The distribution of off-by-one dalton errors is presented in Figure 6. Exact results for cases when off-by-one dalton errors did not occur are presented in the table below in ppm (Da): kDaenvemindMIND8–200.0693
(0.0009)0.0670 (0.0009)20–400.0474
(0.0014)0.0549 (0.0016)40–600.0393
(0.0019)0.0478 (0.0023)For tests on spectra with visible isotopic envelopes, we used 550 spectra segments. Note that the average mass of insulin is a bit below the mass range of the training set, but it still provides accurate results. MAE was equal to 0.0338 Da (2.57 ppm). In 547 cases (99.5%), the off-by-one dalton errors did not occur. If we consider only these cases, MAE drops to 0.0286 Da (2.06 ppm). Only 400 used spectra segments fit in the MIND mass range. They never got off-by-one dalton errors, while envemind had 2 such errors out of 400. MAE when off-by-one dalton errors did not occur were 0.0349 Da (2.06 ppm) for envemind and 0.0278 Da (1.64 ppm) for MIND.

Finally, we tested envemind on spectra with lower quality than expected, 1500 spectra segments. Off-by-one dalton errors occurred in 1258 cases. The MAE was 0.0367 Da (2.53 ppm) for cases that did not occur. For MIND, the number of off-by-one dalton errors was 799, with MAE of 0.0280 Da (2.13 ppm) for the remaining cases.

Let us remind about two essential assumptions of the envemind algorithm. The first is the quality of data. As can be noticed, its performance grows as data quality grows. Therefore, it should be used for the best quality spectra, where the probability of off-by-one dalton error has to be minimized. The algorithm also has a wide mass range, which is another advantage over other algorithms. Since MIND for its prediction requires only mass on the most abundant peak, it is a useful tool for noised spectra, especially when only a very few peaks are visible. The second assumption is that the envemind algorithm only works on spectra of a single molecule. Hence, the molecule under study has to be isolated in a mass spectrometer, or deconvolution can be performed using appropriate algorithms like masserstein.^14^

Let us discuss a bit more about simulating theoretical spectra for experimental ones. In this work, we described a construction method that provides good prediction in a short computational time. Note that, for this purpose, we used Wasserstein distance. However, there are more measures to compare spectra, and new ones are under research. Unfortunately, modern measures require too much computational time and sometimes lead to mistakes to be avoided. On the other hand, the envemind algorithm is very flexible, and applying new measures will be easy when they appear. That may improve prediction and extend utility to low-resolution spectra too.

Also, the whole procedure of obtaining a simulated spectrum can be replaced as new measures appear. We developed an alternative method, which we expect to be more accurate. However, due to long computational time of modern measures, the method is awkward. The method compares many theoretical spectra of substances with similar average mass to an experimental spectrum instead of constructing simulated spectrum from scratch. A detailed description of this approach is attached in the Supporting Information with the use of masserstein measure.

We argue that the monoisotopic peak mass prediction can be based on such artificial but, at the same time, well-fitted spectrum. Notice that the algorithm fits the inherently noisy experimental spectra, and only certain peaks are well-visible. Therefore, the obtained spectrum can differ from the true one. However, it should have the same average mass and variance, which is essential. On the other hand, one may ask if we can determine monoisotopic mass directly from the matched spectrum. Tests show that, on our data, both approaches gave almost identical results. However, the linear model provides a safe solution since the model was trained on actual proteins.

In the end, we would like to highlight innovative aspects of this work. Under the envemind algorithm stands the idea of mathematical exploring of molecule space, where every 5-dimensional integer point is considered as chemical formula and theoretical spectrum simultaneously. Such a point of view lets us elaborate vectors that added to a chemical formula change (or do not) individual features of the spectrum, like average mass or variance. We know where the optimal solution should be looked for in the space with this knowledge.

## Supporting Information Available

Mathematical details of the envemind algorithm and description of alternative way for matching theoretical spectra to experimental ones (PDF)
