# Supplementary material for: Envemind: Accurate Monoisotopic Mass Determination Based On Isotopic Envelope
Source: J Am Soc Mass Spectrom. 2022 Oct 12;33(11):2063–9. doi: 10.1021/jasms.2c00176 (PMC9634886; doi:10.1021/jasms.2c00176)

Supporting Information
Envemind: Accurate Monoisotopic Mass Determination Based On Isotopic Envelope

Piotr Radziński,*,† Dirk Valkenborg,‡ Michał Piotr Startek,†,¶ and Anna Gambin†

† *Institute of Informatics, University of Warsaw, 00-927 Warsaw, Poland*

‡ *Interuniversity Institute of Biostatistics and Statistical Bioinformatics, Hasselt University, BE3500 Hasselt, Belgium*

¶ *Institute of Immunology, University Medical Center of the Johannes-Gutenberg University Mainz, Mainz 55131, Germany*

e-mail: pmradzinski@mimuw.edu.pl

Supporting Information Available

Linear models calibration

Linear model (1) that performs initial prediction of monoisotopic mass, gave us mean absolute error (MAE) equal to 0.1383 in 10-fold cross-validation test. Linear model (2) that explains $\hat{\zeta}$, gave $1.3102 \cdot 10^{-4}$ MAE in 10-fold cross-validation test.

$\hat{\Delta}$ computation note

Recall that the real part of the logarithm of normalized complex number is equal to zero, and the normalization does not change the angle of complex number (i.e. imaginary part either). Therefore the normalization is not required in the computations.

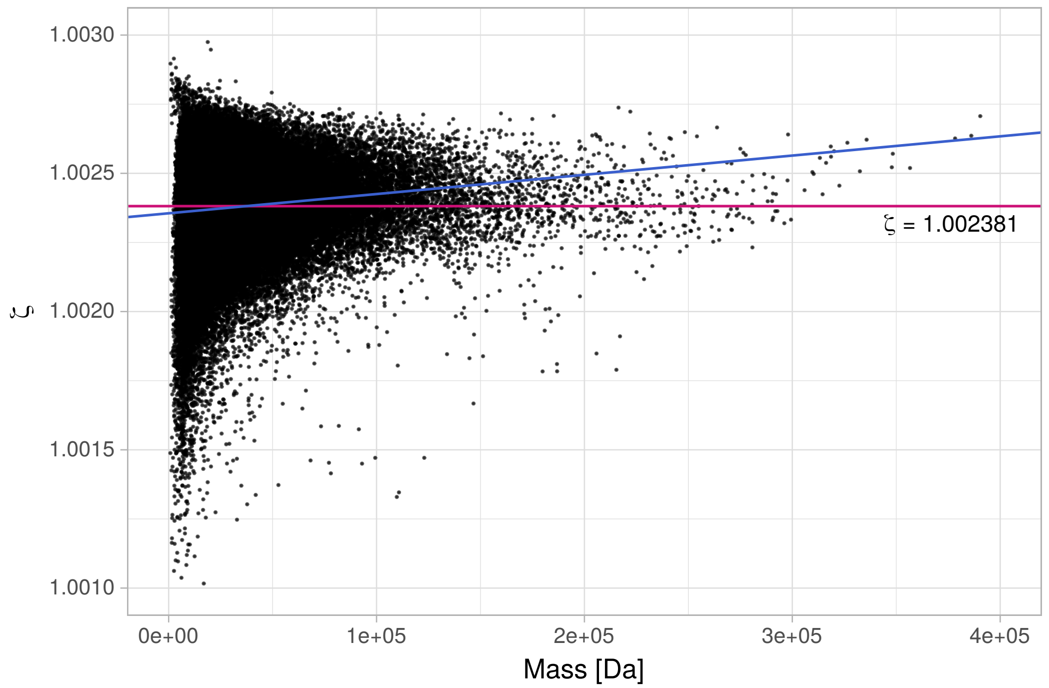

Figure S1: Average theoretical mass versus ζ that minimize variance on complex circle plot. Almost 80'000 randomly chosen proteins from Uniprot database are presented. Pink line presents average ζ equal to 1.002381, blue line is adjusted by linear regression.

Correction of ppm mean error

Let us assume, that we have positive dependent variables Y_i and their predictions $\hat{Y}_i$. Mean error in parts per million (ppm) is defined as

$$c = \operatorname*{mean}_i \left[\frac{Y_i - \hat{Y}_i}{Y_i} \cdot 10^6 \right]. \tag{3}$$

In case $c \neq 0$, i.e. when a distribution of ppm errors isn't distributed around 0, we can improve prediction by moving the distribution to mean 0. To do that, we look for such z, that fulfill following equation

$$\frac{\hat{Y}_i + z}{Y_i} \cdot 10^6 = \frac{\hat{Y}_i}{Y_i} \cdot 10^6 - c \implies z = -\frac{cY_i}{10^6}. \tag{4}$$

Note, that usually during prediction of $\hat{Y}_i$ we don't know value Y_i. However, in our work, we can use initial prediction $\hat{M}_{\text{mono}}$ to fix distribution of $\hat{\hat{M}}_{\text{mono}}$ for which $c \approx 0.12$.

Matching theoretical spectra – alternative approach

The approach described here uses advanced measures to compare theoretical and experimental spectra to find the most fitting spectrum on a given set of potential chemical formulas. Unfortunately, such an approach requires much computational time and have some minor issues. Hence, we decided to use a far faster method described in the manuscript for general use. However, as the development of such measures is a dynamic field, we believe that this approach can become more accurate in the future, as new measures will be proposed. So far, we found that the measure that is fastest and has the most accurate fit was `masserstein` proposed and implemented in [14]. As checking the quality of approximation by a given single formula takes a few seconds, we have to explore only a small subset of the huge search space of potential chemical formulas to search for the best fitting chemical formula. Our search algorithm have two steps:

(A) find a preliminary formula with an average mass close to average experimental mass,

(B) fit the shape (especially variance) of the simulated spectrum to the shape of the experimental spectrum.

In the sequel, we sketch only the basic idea of the 2-step approximation algorithm. Mathematical details can be found in the section below.

Step (A). At the beginning, from the experimental spectrum, we pick up the most abundant peak's mass because it is easily accessible and should be close to the average mass. Then, we use the selected mass to construct the average protein called *averagine*, a newer version of molecule proposed in [7]. Next, starting with the obtained chemical formula, we pick points in the space of chemical formulas that can be generated by adding the combination of two vectors: first, that is responsible for changing the average mass, and second, responsible

for changing the variance of the spectrum without changing the average mass. Then, in all chemical formulas resulting from the described procedure, we change the number of hydrogen atoms to have spectra into the area concentrated ca. 2.5 Da around the most abundant mass. Finally, we apply the concept of optimal transport implemented in `masserstein` algorithm to find the best chemical formula. Our experimental spectrum is compared to all generated formulas, and the Wasserstein (optimal transport) distance between all pairs are calculated, yielding for each formula the estimation of how many peaks in the spectrum correspond to it and how many are classified as noise (i.e. do not belong to optimal transport scenario). The formula having the smallest proportion of the noise is considered to have the most similar average mass and variance to the protein's theoretical spectrum.

Step (B). The main idea of this step is analogous to step (A): we generate a set of chemical formulas and select the optimal one. As a starting point, we take averagine with mass taken from the previous step best fitting formula, and we modify it to have adequate variance. Now, to generate chemical formulas, we use vectors that correspond to directions invariant for the spectrum's average mass and variance. In this case, the only changes of the variance and average mass w.r.t. the original formula are caused by rounding and adding hydrogen atoms. For all generated chemical formulas, we estimate the proportion of experimental spectrum classified as noise by `masserstein` algorithm. The chemical formula that obtains the highest proportion of experimental spectrum is intended to have its theoretical spectrum very close to the experimental one. Therefore, we use it to predict the monoisotopic peak as described in the previous sections.

In Fig. 2, we present two examples of matching theoretical spectra to the experimental one. In the left column, the fitted spectrum and theoretical of truth protein are very similar. Because in most of the cases we obtain such similarity, results on high-resolution experimental spectra are close to those on theoretical ones (96.6% of close-to-0Da errors). On the other hand, in the right column of the plot, we show case, when the experimental spectrum is shifted a bit, thus matched spectrum is shifted too. In this case, +1 Da error occurred. Let

us note that both presented spectra are high quality. As quality goes down, `masserstein` algorithm measure fitting peaks in wrong places is more probable. Again, we believe that future measures will deal with the problem of shift of peaks (i.e., places of peaks will be more important than their intensities).

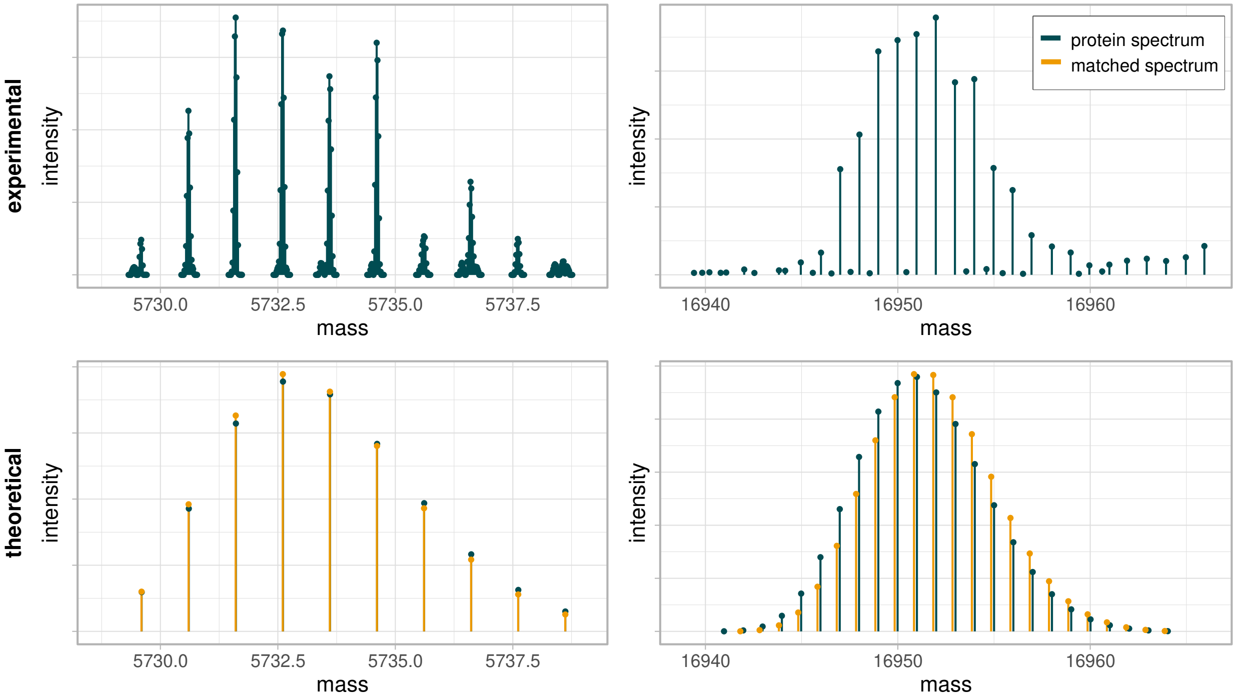

Figure S2: Two examples of matched spectra. In each column, experimental and theoretical spectra of insulin and myoglobin are presented, respectively (blue). In the second row, theoretical spectra that were matched to the experimental one are imposed on (orange). Note that in the right column, we present a badly matched spectrum for which off-by-dalton error occurred.

We tested `envemind` algorithm on 550 high-resolution spectra. For 534 cases (97.1%), the predicted monoisotopic mass was no further than 0.5 Da from the truth value.

We can add voting to this approach to minimize the risk of a shifted match. Instead of picking the best-matched spectrum, we can pick, e.g. 5 with best match scores. Then, run prediction on every one of them and compute the average monoisotopic mass from the area where most of them. Unfortunately, this improvement is less effective than could be expected. It gave slightly better results, but with the same amount of ± 1 Da errors. Usually, all matches are in a similar area, and thus it does not deal with them. However, it lowered

the amount of >0.2 Da errors from 23/550 to 17/550.

Mathematical description

Here we describe how we find a chemical formula that approximates a given experimental spectrum in a more mathematical way than it is described above. We denote the space of chemical formulas by $\mathcal{V}$ that is equal to $\mathbb{N}^5$. To start, we take the highest peak that we can find in the experimental spectrum. Then, we calculate averagine protein with the given mass, where coordinates can be real numbers, not necessarily natural. Then, we generate the first set of potential chemical formulas that are intended to find a formula with close average mass but not necessarily the same shape:

$$\mathcal{H}_* = \{\vartheta \in \mathcal{V} : \vartheta = \mathrm{crl}(A_{\mathrm{abu}} + i \cdot A_{\mathrm{norm}} + j \cdot V),\ i, j \in \{-n_1, \ldots, n_1\}\},$$

where

- $\mathrm{crl}(x)$ - function that rounds x to closest point in $\mathcal{V}$ (in $||.||_2$ norm), and then add such integer of hydrogen atoms, to have mass closest possible to starting point;
- n_1 - parameter responsible for set size. Bigger n_1 gives better fit of spectrum but extends time of computations. We use n_1 equal to around 5.
- A_{abu} - averagine vector with mass equal to highest peak's mass in experimental spectrum;
- A_{norm} - averagine vector normed to have length 1, i.e. equal to (0.5230, 0.8255, 0.1439, 0.1550, 0.0037);
- V - vector responsible for fastest change of variance without changing average mass. It is easy to prove that vectors of chemical element's atoms mass and variance (in table below) are also directions where given feature grows fastest.

Element	C	H	N	O	S
Average Mass	12.0108	1.0079	14.0067	15.9994	32.0649
Variance	0.0107	0.0001	0.0036	0.0086	0.1700

Both features are linear, i.e. can be added and multiplied, e.g. H_2O have average mass 18.0152 and variance 0.0088. To obtain vector that change variance as fast as possible, but without changing average mass we use following lemma.

Lemma 0.1. *Let* $f(x) = \langle v, x\rangle$ *and*

$$\Lambda = \{x \in \mathbb{R}^n : \langle x, x\rangle = 1, \langle a, x\rangle = 0\},$$

for given $a, v \in \mathbb{R}^n$*. Then extrema of* $f(x)$ *on* Λ *are reached in*

$$x = \frac{v - \text{proj}_a v}{||v - \text{proj}_a v||} \quad \text{and} \quad x = -\frac{v - \text{proj}_a v}{||v - \text{proj}_a v||}.$$

Proof. Since Λ is compact manifold, we can use method of Lagrange multipliers to find extrema. We define Lagrange function

$$L(x, \lambda_1, \lambda_2) = \langle v, x\rangle + \lambda_1(\langle x, x\rangle - 1) + \lambda_2\langle a, x\rangle,$$

which gives us following system of equations:

$$\begin{cases} \langle x, x\rangle - 1 = 0 \\ \langle a, x\rangle = 0 \\ v + 2\lambda_1 x + \lambda_2 a = 0 \end{cases}$$

By third equation we have

$$x = \frac{-v - \lambda_2 a}{2\lambda_1}.$$

We calculate λ_1 from the first equation and obtain $\lambda_1 = \pm\frac{1}{2}||v + \lambda_2 a||$. Hence, we can

write

$$x = \mp \frac{v + \lambda_2 a}{||v + \lambda_2 a||}.$$

From first equation we obtain $\lambda_2 = -\frac{\langle a,v \rangle}{\langle a,a \rangle}$ what gives us final result

$$x = \mp \frac{v - \frac{\langle a,v \rangle}{\langle a,a \rangle} a}{||v - \frac{\langle a,v \rangle}{\langle a,a \rangle} a||} = \mp \frac{v - \mathrm{proj}_a v}{||v - \mathrm{proj}_a v||}.$$

□

In our case a is vector of average masses, v is vector of variances as in table above. Finally $V = (-0.3430, -0.0372, -0.4960, -0.5184, 0.6050)$.

Because stochastic noise makes that highest peak can differ from theoretical highest peak, we add up to ± 2 hydrogen atoms and we obtain

$$\mathcal{H}_1 = \{\vartheta \in \mathcal{V} : \vartheta = \vartheta_* + (0, h, 0, 0, 0),\ \vartheta_* \in \mathcal{H}_*,\ h \in \{-2, \ldots, 2\}\}.$$

We check which formula has the best proportion to the experimental spectrum on this set, as described in the manuscript. Form a formula with the highest proportion score, we take average mass and variance and use them to construct a new starting point. We create averagine with a given mass, and then we change variance (by adding V vector that does not change average mass) to be adequate. We denoted the new starting point by B. This time potential points are intended to fit the shape of the spectrum too:

$$\mathcal{H}_2 = \{\vartheta \in \mathcal{V} : \vartheta = \mathrm{crl}(B + i \cdot S_1 + j \cdot S_2 + k \cdot S_3),\ i, j, k \in \{-n_2, \ldots, n_2\}\},$$

where S_i are three solutions of system of equations

$$\begin{cases} 0.2979\vartheta_c + 0.0250\vartheta_h + 0.3474\vartheta_n + 0.3968\vartheta_o + 0.7953\vartheta_s = 0 & \text{Average Mass} \\ 0.0630\vartheta_c + 0.0007\vartheta_h + 0.0211\vartheta_n + 0.0504\vartheta_o + 0.9965\vartheta_s = 0 & \text{Variance} \end{cases}$$

Coefficients of the system are normalized values from table above, what results that solutions S_i do not change average mass and variance. As previously, n_2 parameter control size of the set, we recommend to use n_2 equal to around 10-12. Chemical formula with highest proportion score fits experimental spectrum best.

Graphical TOC Entry

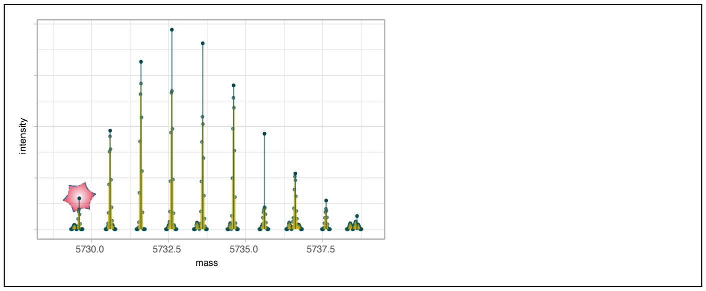

Supplement: Supplementary file 1 — js2c00176_si_001.pdf [file js2c00176_si_001.pdf]
